# In vitro activities of 11 fluoroquinolones against 816 non-typhoidal strains of *Salmonella enterica *isolated from Finnish patients with special reference to reduced ciprofloxacin susceptibility

**DOI:** 10.1186/1476-0711-4-12

**Published:** 2005-09-05

**Authors:** Pirkko Kotilainen, Susa Pitkänen, Anja Siitonen, Pentti Huovinen, Antti J Hakanen

**Affiliations:** 1 Antimicrobial Research Laboratory, Department of Bacterial and Inflammatory Diseases, National Public Health Institute, Turku, Finland; 2 Department of Medicine, Turku University Hospital and Turku University, Turku, Finland; 3 Enteric Bacteria Laboratory, Department of Bacterial and Inflammatory Diseases, National Public Health Institute, Helsinki, Finland

## Abstract

**Background:**

The number of *Salmonella *strains with reduced susceptibility to fluoroquinolones has increased during recent years in many countries, threatening the value of this antimicrobial group in the treatment of severe salmonella infections.

**Methods:**

We analyzed the in vitro activities of ciprofloxacin and 10 additional fluoroquinolones against 816 *Salmonella *strains collected from Finnish patients between 1995 and 2003. Special attention was focused on the efficacy of newer fluoroquinolones against the *Salmonella *strains with reduced ciprofloxacin susceptibility.

**Results:**

The isolates represented 119 different serotypes. Of all 816 *Salmonella *strains, 3 (0.4%) were resistant to ciprofloxacin (MIC ≥ 4 μg/ml), 232 (28.4%) showed reduced susceptibility to ciprofloxacin (MIC ≥ 0.125 – 2 μg/ml), and 581 (71.2%) were ciprofloxacin-susceptible. The MIC_50 _and MIC_90 _values of ciprofloxacin for these strains were 0.032 and 0.25 μg/ml, respectively, being lower than those of the other fluoroquinolone compounds presently on market in Finland (ofloxacin, norfloxacin, levofloxacin, and moxifloxacin). For two newer quinolones, clinafloxacin and sitafloxacin, the MIC_50 _and MIC_90 _values were lowest, both 0.016 and 0.064 μg/ml, respectively. Moreover, clinafloxacin and sitafloxacin exhibited the lowest MIC_50 _and MIC_90 _values, 0.064 and 0.125 μg/ml, against the 235 *Salmonella *strains with reduced susceptibility and strains fully resistant to ciprofloxacin.

**Conclusion:**

Among the registered fluoroquinolones in Finland, ciprofloxacin still appears to be the most effective drug for the treatment salmonella infections. Among the newer preparations, both clinafloxacin and sitafloxacin are promising based on in vitro studies, especially for strains showing reduced ciprofloxacin susceptibility. Their efficacy, however, has not been demonstrated in clinical investigations.

## Background

Fluoroquinolones generally have a good in vitro and clinical activity against isolates of the *Salmonella *species [1]. Although the Clinical and Laboratory Standards Institute (CLSI; formerly the National Committee for Clinical Laboratory Standards, NCCLS) guidelines recommend the MICs of ≤1 and ≥4 μg/ml as respective breakpoints of susceptibility and resistance to ciprofloxacin [2], it is now commonly accepted that *Salmonella *isolates with MICs between ≥0.125 and 2 μg/ml are characterized by reduced susceptibility i.e. low-level resistance to fluoroquinolones. The selection of MICs ≥0.125 μg/ml as a breakpoint of reduced ciprofloxacin susceptibility is justified based on histogram and scatterblot analyses combined with sequencing data [3-8]. This selection is further supported by reports on several treatment failures with ciprofloxacin and other fluoroquinolones in patients with infections caused by *Salmonella *strains showing reduced susceptibility to fluoroquinolones [9-17].

It is of concern that in recent years, the number of *Salmonella *isolates with reduced fluoroquinolone susceptibility has increased in many countries, including countries of the European Union [6,18-21]. In Finland, reduced fluoroquinolone susceptibility emerged between 1995 and 1997 among *Salmonella *isolates of domestic origin [21], and a significant increase in the annual proportion of reduced fluoroquinolone susceptibility was observed between 1995 and 1999 among both the domestic and foreign isolates [5].

Several fluoroquinolone preparations are on market all over the world, while a number of new compounds are presently being developed or undergoing clinical studies. The purpose of the present study was to determine the in vitro activities of various older and newer fluoroquinolones towards the *Salmonella *species, focusing special attention on the isolates with reduced ciprofloxacin susceptibility. In so doing, we determined the susceptibilities of 816 epidemiologically unrelated *Salmonella *isolates to ciprofloxacin and 10 additional fluoroquinolones including two novel extended-spectrum compounds: clinafloxacin and sitafloxacin.

## Methods

### Salmonella strains

Antimicrobial susceptibility of *Salmonella enterica *isolates has been surveyed in the National Public Health Institute, Finland, since 1995 by analyzing yearly 200–400 strains collected from Finnish patients seeking medical assistance for gastroenteritis. Starting in January each year, we consecutively collect 100–200 foreign strains and 100–200 domestic (i.e., Finnish) strains for susceptibility testing. An isolate is designated as of foreign origin, if the patient has reported travel abroad during one month before the specimen day. All other isolates are designated as of domestic origin. Epidemiological information regarding potential travelling and the travel destination is collected from the forms which accompany each isolate sent to the Enteric Bacteria Laboratory of the National Public Health Institute, Helsinki, which serves as the National *Salmonella *Reference Centre in Finland.

We included in this study a total of 816 clinical *Salmonella *strains collected between 1995 and 2003 during the annual surveys. All strains were isolated from stool. Of these strains, 365 were designated as of domestic origin and 451 as of foreign origin. The strains were considered to be epidemiologically unrelated based on their recovery from distinct sources. For each *Salmonella *outbreak recognized, only one isolate representing the epidemic strain was included. The *Salmonella *collection consisted of 119 different serotypes. All strains belonged to non-typhoidal *Salmonella enterica*. The most prevalent serotypes were *S*. Enteritidis, *S*. Typhimurium, and *S*. Hadar, accounting for 27.2%, 19.0%, and 7.1% of the isolates, respectively.

### Susceptibility testing

The minimum inhibitory concentrations (MIC) of the isolates were determined by the standard agar plate dilution method according to the NCCLS guidelines [22]. Mueller-Hinton II agar (BBL, Becton Dickinson and Company, Cockeysville, Md.) was used as the culture medium. Altogether 11 fluoroquinolone compounds were analyzed. Reagent powder of each of these agents was provided by its manufactorer: ciprofloxacin (Bayer, Wuppertal, Germany), clinafloxacin (Pfizer, Ann Arbor, MI, Unites States), enrofloxacin (Bayer, Elberfeld, Germany), gatifloxacin (Grunenthal BHBH, Aachen, Germany), gemifloxacin (GlaxoSmithKline, Worthing, United Kingdom), levofloxacin (Hoechst Marion Roussel, Romainville Cedex, France), lomefloxacin (Sigma, St. Luis, MO, United States), moxifloxacin (Bayer, Wuppertal, Germany), norfloxacin and ofloxacin (Sigma, Steinheim, Germany), and sitafloxacin (Daiichi Pharmaceuticals, Tokyo, Japan). The *Salmonella *isolates were also tested for susceptibility to nalidixic acid.

Of the fluoroquinolone compounds analyzed, ciprofloxacin, ofloxacin, norfloxacin, levofloxacin, and moxifloxacin are presently on market in Finland.

*Staphylococcus aureus *ATCC 29213, *Escherichia coli *ATCC 25922, *E. coli *ATCC 35218, and *Pseudomonas aeruginosa *ATCC 27853 were used as controls in susceptibility testing.

### Data analysis

The susceptibility data were analyzed using the WHONET5 computer program (available from ).

## Results

Of all 816 *Salmonella *strains, 3 (0.4%) were resistant to ciprofloxacin (MIC ≥ 4 μg/ml), 232 (28.4%) showed reduced susceptibility to ciprofloxacin (MIC ≥ 0.125 – 2 μg/ml), and 581 (71.2%) were ciprofloxacin-susceptible. Of the 451 foreign *Salmonella *strains, 2 (0.4%) were ciprofloxacin-resistant and 193 (42.8%) showed reduced susceptibility to ciprofloxacin. Of the 365 domestic *Salmonella *strains, 1 (0.3%) was ciprofloxacin-resistant and 39 (10.7%) showed reduced susceptibility to ciprofloxacin.

**Figure 1:**
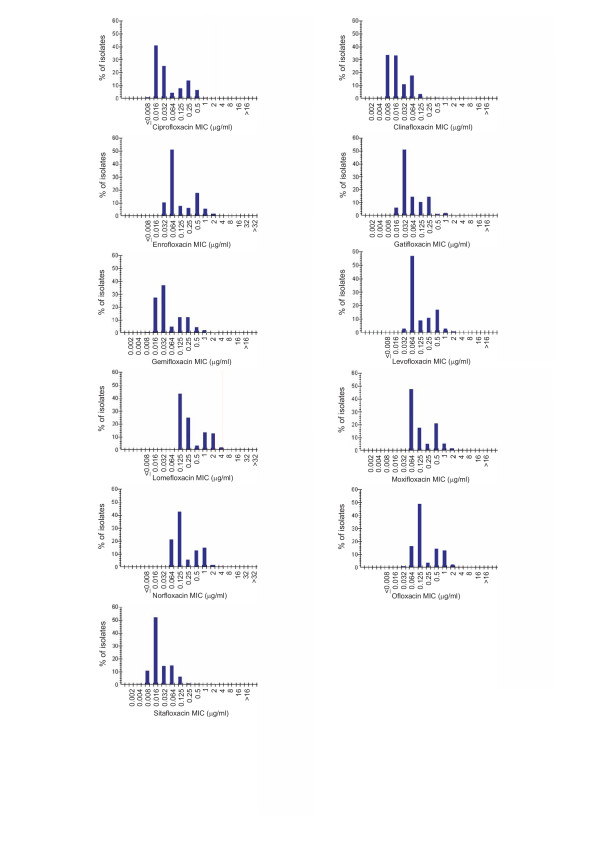
**MIC histograms of fluoroquinolones**. Minimum inhibitory concentration (MIC) histograms of 11 fluoroquinolones against 816 *Salmonella *strains collected from Finnish patients between January 1995 and January 2003.

For all 816 strains, the MIC_50 _and MIC_90 _values of ciprofloxacin were 0.032 and 0.25 μg/ml, respectively (Table 1). For ofloxacin, levofloxacin, norfloxacin, and moxifloxacin, the MIC_50 _values varied between 0.064 and 0.125 μg/ml; and the MIC_90 _values, between 0.5 and 1 μg/ml. The MIC_50 _and MIC_90 _values of enrofloxacin, lomefloxacin, gatifloxacin, and gemifloxacin were similar to or higher than those of ciprofloxacin. For both clinafloxacin and sitafloxacin, the MIC_50 _and MIC_90 _values were lower than for the other agents tested, 0.016 and 0.064 μg/ml, respectively. The histograms illustrating the MICs of the fluoroquinolones studied are presented in Figure 1. The MIC_50 _and MIC_90 _values of nalidixic acid were 4 and 512 μg/ml, respectively (range, <0.5 to >512 μg/ml).

**Table 1:** MICs of 11 fluoroquinolones for 816 non-typhoidal strains of *Salmonella enterica *isolated from Finnish patients between 1995 and 2003

	MIC (μg/ml)			
	
Fluoroquinolone	Number of isolates	MIC_50_	MIC_90_	Range
Ciprofloxacin	816	0.032	0.25	0.008 – 16
Clinafloxacin	816	0.016	0.064	0.002 – 2
Enrofloxacin	808	0.064	0.5	0.032 – >32
Gatifloxacin	816	0.032	0.25	0.002 – 8
Gemifloxacin	815	0.032	0.25	0.002 – >16
Levofloxacin	808	0.064	0.5	≤0.008 – 16
Lomefloxacin	807	0.25	2	0.064 – >32
Moxifloxacin	816	0.125	0.5	0.008 – >16
Norfloxacin	816	0.125	1	0.016 – >32
Ofloxacin	816	0.125	1	0.016 – >16
Sitafloxacin	816	0.016	0.064	0.002 – 2

The most prevalent serotypes among the 235 *Salmonella *strains with reduced susceptibility or resistance to ciprofloxacin were *S*. Hadar, *S*. Enteritidis, and *S*. Virchow accounting for 22.6%, 20.9%, and 12.3% of the strains, respectively. For these 235 strains, the MIC_50 _values of ofloxacin, levofloxacin, norfloxacin and moxifloxacin varied between 0.5 and 1 μg/ml; and the MIC_90 _value was 1 μg/ml for all (Table 2). The MIC_50 _and MIC_90 _values were lowest, 0.064 and 0.125 μg/ml, for both clinafloxacin and sitafloxacin. For the additional 4 fluoroquinolones examined, the MIC_50 _varied between 0.25 and 2 μg/ml; and the MIC_90 _values, between 0.5 and 2 μg/ml. The MIC_50 _and MIC_90 _values of nalidixic acid for these strains were 512 and >512 μg/ml, respectively (range, 4 to >512 μg/ml).

**Table 2:** MICs of 11 fluoroquinolones for 235 non-typhoidal strains of *Salmonella enterica *with reduced ciprofloxacin susceptibility^1 ^or ciprofloxacin resistance^2 ^isolated from Finnish patients between 1995 and 2003

	MIC (μg/ml)			
	
Fluoroquinolone	Number of isolates	MIC_50_	MIC_90_	Range
Ciprofloxacin	235	0.25	0.5	0.125 – 16
Clinafloxacin	235	0.064	0.125	0.008 – 2
Enrofloxacin	233	0.5	1	0.032 – >32
Gatifloxacin	235	0.25	0.5	0.032 – 8
Gemifloxacin	235	0.25	0.5	0.064 – >16
Levofloxacin	233	0.5	1	0.064 – 16
Lomefloxacin	232	2	2	0.25 – >32
Moxifloxacin	235	0.5	1	0.25 – >16
Norfloxacin	235	1	1	0.25 – >32
Ofloxacin	235	1	1	0.125 – >16
Sitafloxacin	235	0.064	0.125	0.016 – 2

^1^Ciprofloxacin MIC ≥0.125 – 2 μg/ml

^2^Ciprofloxacin MIC ≥4 μg/ml

**Figure 2:**
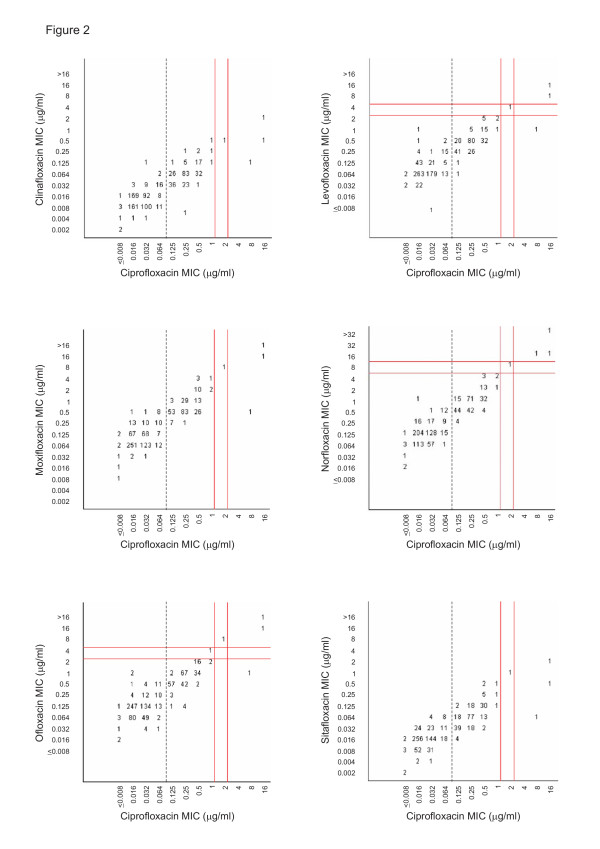
**Scattergrams correlating the MICs of ciprofloxacin to those of other fluoroquinolones**. Scattergrams for 816 *Salmonella *strains correlating the minimum inhibitory concentrations (MIC) of ciprofloxacin to those of clinafloxacin, levofloxacin, moxifloxacin, norfloxacin, ofloxacin, and sitafloxacin. The numbers within the graphs indicate the numbers of *Salmonella *strains. The vertical solid lines indicate the Clinical and Laboratory Standards Istitute (CLSI) breakpoint recommendations for susceptibility and resistance, respectively, to ciprofloxacin (MIC ≤1 and ≥4 μg/ml); and the vertical dashed lines, the breakpoint for reduced susceptibility (MIC ≥0.125 μg/ml). The horizontal solid lines indicate the respective CLSI breakpoint recommendations to those fluoroquinolones for which such recommendations are available: ofloxacin (MIC ≤2 and ≥8 μg/ml); norfloxacin (MIC ≤4 and ≥16 μg/ml); and levofloxacin (MIC ≤2 and ≥8 μg/ml).

The scattergrams correlating the MICs of ciprofloxacin to those of clinafloxacin, levofloxacin, moxifloxacin, norfloxacin, ofloxacin, and sitafloxacin for the 816 *Salmonella *strains are presented in Figure 2. These pictures illustrate that there is a distinct correlation between the ciprofloxacin susceptibility and the susceptibility to other fluoroquinolones. The MICs of levofloxacin, moxifloxacin, norfloxacin, and ofloxacin were generally higher than those of ciprofloxacin, but for each, the fully susceptible population was separate from the population with reduced susceptibility. Of the 232 *Salmonella *strains with reduced ciprofloxacin susceptibility, the MICs of levoxacin, moxifloxacin, norfloxacin, and ofloxacin were 1 or 2 dilution steps higher than those of ciprofloxacin. In contrast, the MICs of clinafloxacin and sitafloxacin were generally 2 dilution steps lower than those of ciprofloxacin.

## Discussion

Of all 816 *Salmonella *strains included in the present study, 28.8% showed resistance or reduced susceptibility to ciprofloxacin. The MIC_50 _and MIC_90 _values of ciprofloxacin for these isolates were 0.032 and 0.25 μg/ml, respectively, being similar to or lower than those of all older fluoroquinolone compounds studied. Thus, we show that ciprofloxacin still is the most effective fluoroquinolone drug registered in Finland for the treatment of salmonellosis. In the entire *Salmonella *collection, the MIC_50 _and MIC_90 _values were lowest for two new quinolones: clinafloxacin and sitafloxacin. Moreover, clinafloxacin and sitafloxacin exhibited the lowest MIC_50 _and MIC_90 _values for the 235 ciprofloxacin-resistant *Salmonella *strains or strains showing reduced ciprofloxacin susceptibility. Based on these in vitro results, both clinafloxacin and sitafloxacin appear promising drugs for the treatment of salmonella infections, but their efficacy has not been demonstrated in clinical investigations.

The introduction of fluoroquinolones in the 1980's had an almost revolutionary effect on the treatment of salmonellosis, since they offered an effective per oral alternative to treat clinical infections as well as to eradicate long-term carriage. So far, the emergence and rapid increase of reduced fluoroquinolone susceptibility in non-typhoidal salmonellas has not led to major consequences, since these microbes characteristically cause gastroenteritis, for which antimicrobial treatment is not always indicated. The situation is totally different in invasive salmonella infections, in which administration of an effective antimicrobial agent is vitally important. If such an infection is caused by a *Salmonella *strain with reduced fluoroquinolone susceptibility, treatment with a fluoroquinolone compound may not be a safe alternative. There are several reports showing that the use of fluoroquinolones in infections caused by *Salmonella *isolates with reduced susceptibility may lead to treatment failures [6-17]. The majority of the cases have involved *Salmonella enterica *serotype Typhi [8,10-12,15]. In non-typhoidal salmonellosis, the causative strain has often been initially fully susceptible, but after fluoroquinolone treatment failure, the MICs for these strains have been ≥0.125 μg/ml [13,14,17].

Clinafloxacin and sitafloxacin are the most interesting fluoroquinolone compounds studied here, since earlier data on their activity against the *Salmonella *species are limited. We found only one previous study, in which the in vitro activity of sitafloxacin against 326 *Salmonella *isolates was tested, with a finding that its activity was equal to or slightly better than that of ciprofloxacin [23].

One of the main purposes of the present study was to analyze the in vitro activities of these two novel fluoroquinolone compounds towards *Salmonella *isolates with reduced ciprofloxacin susceptibility. Our results show that the MIC_50 _and MIC_90 _of clinafloxacin and sitafloxacin against these strains were generally 2 dilution steps lower than those of ciprofloxacin and even 4 to 5 dilution steps lower than some of the older fluoroquinolones. Nevertheless, this does not necessarily mean that they should have a superior in vivo activity. The fluoroquinolone antimicrobial group is characterized by cross resistance, indicating that when a bacterial isolate is resistant to one fluoroquinolone, it is also resistant to other members of the same group [24].

The development of fluoroquinolone resistance most commonly involves a mutation in the quinolone resistance determining region (QRDR) of the *gyr*A gene [4,5,14,25,26]. One single point mutation usually leads to nalidixic acid resistance and reduced fluoroquinolone susceptibility, while additional mutations or accumulation of several resistance mechanisms are needed to produce high-level fluoroquinolone resistance. On this basis, one can assume that in the majority of the *Salmonella *strains with reduced fluoroquinolone susceptibility analyzed here, quinolone resistance is associated with one point mutation. Based on their low MIC values, both clinafloxacin and sitafloxacin may potentially have useful clinical activity against *Salmonella *strains with a single mutation in the *gyr*A gene. It must be borne in mind, however, that having undergone one point mutation, these isolates are potentially inclined to a second mutation, leading to higher MIC values. Thus, it is probable that resistance will develop during fluoroquinolone treatment also against these newer quinolones, despite their initially low MIC values.

It is also of note that the clinical relevance of various MIC values of clinafloxacin and sitafloxacin is not known. Further, no breakpoins of resistance or susceptibility have been given by the CLSI for these newer fluoroquinolone compounds. Neither do we know anything about their potential breakpoints for reduced fluoroquinolone susceptibility.

Many previous studies have focused on in vitro efficacy of clinafloxacin and sitafloxacin on microbes other than salmonellas [23,27-29]. In these studies, the two new fluoroquinolones have had a better activity than the older preparations towards a variety of bacterial species, even including the ciprofloxacin-resistant strains. For example, clinafloxacin exhibited greater activity than older fluoroquinolones against ciprofloxacin-resistant *Klebsiella pneumoniae *and *Enterobacter aerogenes *isolates [27], and sitafloxacin proved superior to the others against ciprofloxacin-resistant isolates of several enterobacterial species [23]. Moreover, a number of studies have demonstrated the clinical efficacy of clinafloxacin in the treatment of systemic infections, including severe skin and soft tissue diseases and infective endocarditis, and in empirical therapy of febrile granulocytopenic patients [30-33]. These data are encouraging and suggest that the clinical efficacy of these novel fluoroquinolones should be tested also in salmonellosis.

The possible clinical usefulness of the two newer quinolones against *Salmonella *strains will also depend on the levels of the drug reaching the infecting organism. It has been shown in previous studies that in general, newer fluoroquinolones have equal or greater bioavailability compared with ciprofloxacin, which varies between 55 to 88% [34]. Limited data suggest that at least 70% of sitafloxacin is absorbed after an oral dose [35]. In one study, the absolute bioavailability of orally administered clinafloxacin was approximately 90% [36]. Thus, it is expected that these drugs will prove effective also in the oral treatment of salmonellosis.

## Conclusion

Among the registered fluoroquinolone compounds in Finland, ciprofloxacin still appears to be the most effective drug for the treatment salmonella infections. Among the newer preparations, both clinafloxacin and sitafloxacin are promising based on in vitro studies, since they exhibited the lowest MIC_50 _and MIC_90 _values for all *Salmonella *isolates as well as for those with reduced fluoroquinolone susceptibility.

## Authors' contributions

PK, SP, PH and AH planned and carried out the design of the study. SP and AH participated in laboratory studies and interpretation of the data. AS participated in the collection of *Salmonella *isolates and data, and was responsible for serotyping of *Salmonella *isolates. PK and AH wrote the first draft of the manuscript. All authors had intellectual contribution, and all read and approved the final manuscript.
